# Fibrosarcomas of the Paranasal Sinuses: A Systematic Review

**DOI:** 10.7759/cureus.27868

**Published:** 2022-08-10

**Authors:** Moneb S Bughrara, Tarek Almsaddi, Jithin John, Brandon Prentice, Jared Johnson, Oswaldo Henriquez, Adam J Folbe

**Affiliations:** 1 Internal Medicine, Oakland University William Beaumont School of Medicine, Rochester Hills, USA; 2 Otolaryngology, Oakland University William Beaumont School of Medicine, Rochester Hills, USA; 3 Otolaryngology - Head and Neck Surgery, Wayne State University School of Medicine, Detroit, USA; 4 Otolaryngology, Emory University, Atlanta, USA; 5 Otolaryngology - Head and Neck Surgery, Beaumont Hospital, Royal Oak, USA

**Keywords:** maxillary sinus neoplasms, sarcoma, sinus oncology, sinus disease, paranasal sinus diseases, fibrosarcoma

## Abstract

Fibrosarcomas are rare, malignant neoplasms of mesenchymal origin. Fibrosarcomas appear to be sporadic, but cases of fibrosarcomas secondary to radiation of nasopharyngeal carcinomas have been reported. Paranasal sinus fibrosarcomas (PNFS) are even rarer with few cases being reported since the 1950s. There have been several retrospective cohort studies examining PNFS; however, to our knowledge, no comprehensive review exists. This review aims to summarize the findings of all published cases of PNFS from the 1950s to the 2020s. We hope that a comprehensive review will assist in accurate and early diagnoses of PNFS, and help guide treatment as early treatment is associated with a favorable prognosis.This systematic review reports results following the Preferred Reporting Items for Systematic Reviews and Meta-Analyses (PRISMA) guidelines. A search was conducted on PubMed, Embase, and Cochrane Library. Studies were screened using established inclusion/exclusion criteria. A total of 26 studies were included for data extraction, and relevant data were collected and analyzed.In our study, the most common study type was case reports (n = 19). The most common presentation for PNFS included male gender (n = 17) with maxillary sinus (n = 57) involvement. Patients commonly presented with complaints of nasal obstruction (n = 15), epistaxis (n = 11), and facial fullness/pain (n = 9). Surgical resection was the mainstay treatment, with the use of chemotherapy or radiation depending on surgical margins and resectability. The diagnosis was commonly made with histological analysis. This review of the literature provides a summary and reference of important presenting factors, elements of diagnosis, and treatment options regarding PNFS to help bring awareness and guide the treatment of such a rare disease. Moving forward, there is a greater need for larger standardized studies that can further complement our findings, as well as more consistent reporting of cases.

## Introduction and background

Fibrosarcomas are rare, malignant neoplasms of mesenchymal origin that comprise 7-10% of all head and neck sarcomas [1]. Fibrosarcoma appears to be sporadic, but cases of fibrosarcoma secondary to radiation of nasopharyngeal carcinomas have been reported [2]. Paranasal sinus fibrosarcomas (PNFS) are even rarer with few cases being reported since the 1950s. There have been several retrospective cohort studies examining PNFS; however, to our knowledge, no complete review exists [3,4].

As with other nasal cavity and paranasal sinus pathologies, PNFS often presents with unilateral nasal obstruction and epistaxis, sometimes being mistaken as a papilloma [1]. Previous reports have found associations with age and gender but reports vary [4,5]. Final staging and diagnosis are based on imaging, histopathology, and immunohistochemistry, with a characteristic herringbone arrangement of fibroblasts being pathognomonic [6].

PNFS is associated with a high risk of local recurrence and a low risk of distant metastasis [3]. Due to this association, PNFS are often treated with local excision with large margins with or without radiotherapy [1]. The extent of resection is also dependent upon the presence or absence of bone invasion. The anatomical site seems to correlate with the prognosis of sarcomas in general, with lesions of extremities having a more favorable outcome than central locations such as the pelvis, head/neck, and rib [2,7]. Prognosis according to paranasal sinus location has not been defined.

This review aims to summarize the findings of all published cases of PNFS from the 1950s to the 2020s. A total of 109 cases from 26 articles were collected from PubMed, Embase, and Cochrane. This review covers study characteristics, presentation of symptoms, location of the tumor, pathological findings, diagnosis, treatment, and complications. We hope that a comprehensive review will assist in accurate and early diagnoses of PNFS, as early treatment is associated with a favorable prognosis.

## Review

Methods

**Figure 1:**
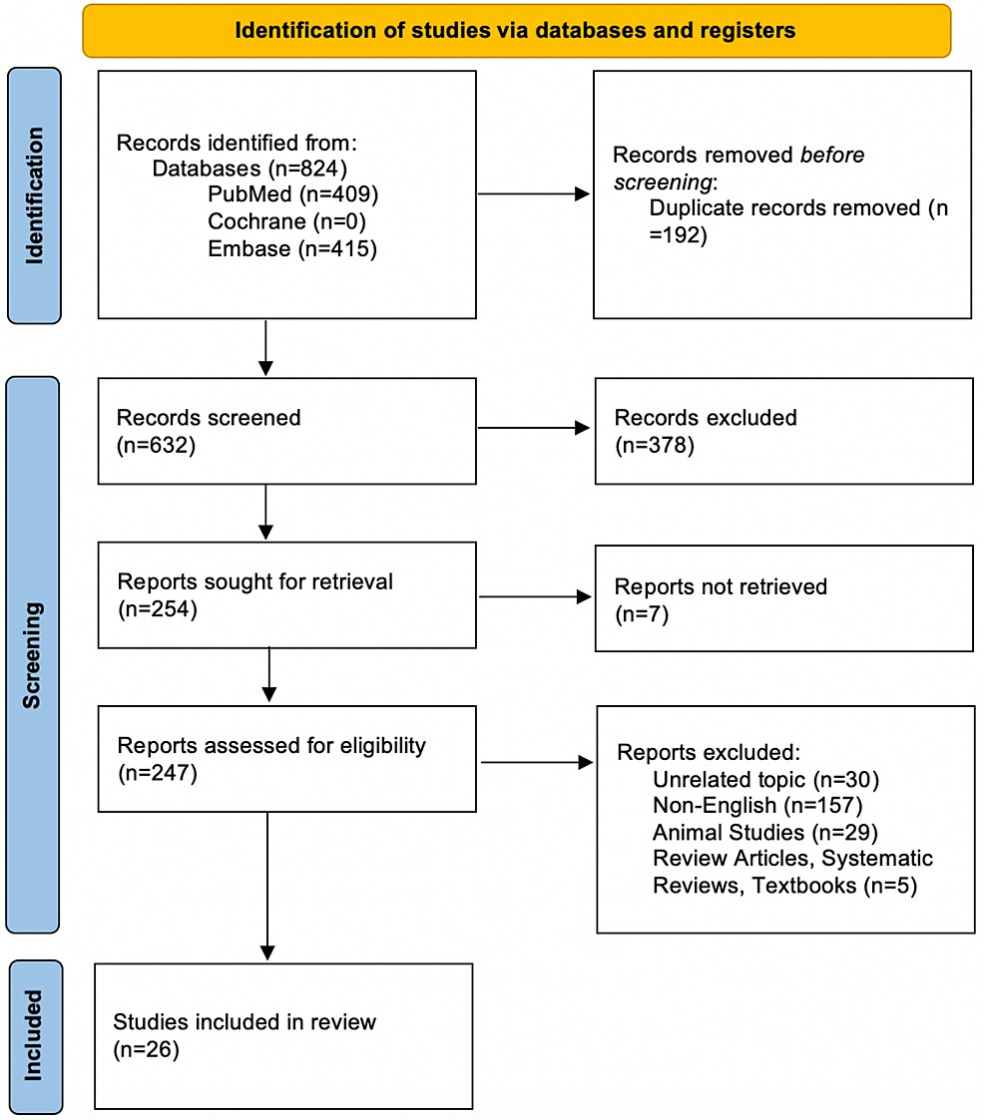
Article selection process based on the Preferred Reporting Items for Systematic Reviews and Meta-Analyses (PRISMA) guidelines

This systematic review reports results following the Preferred Reporting Items for Systematic Reviews and Meta-Analyses (PRISMA) guidelines [8]. A search was conducted on PubMed, Embase, and Cochrane Library on September 25th, 2020 (Appendix). The objective of the study and inclusion/exclusion criteria were documented prior to initiating the study. Figure 1 demonstrates the search strategy for this review. All studies were assigned levels of evidence according to the Oxford Centre for Evidence-Based Medicine (OCEBM) [9].

Study Selection

Titles and abstracts of studies retrieved were screened for inclusion by two independent reviewers, and a separate third reviewer resolved all conflicts. The full text of included studies was subsequently reviewed. Studies were screened for inclusion using the Medical Subject Heading (MeSH) terms related to fibrosarcoma of the paranasal sinuses. Inclusion criteria included studies of patients with fibrosarcomas in the paranasal regions. Articles that were non-English, non-human studies, review articles, books, and studies unrelated to fibrosarcoma of the paranasal sinuses were excluded. Twenty-six studies were included for data extraction.

Data Extraction

Data extraction was conducted using a Microsoft Excel spreadsheet (Microsoft Corporation, Redmond, WA). Patient demographics were collected, such as age and gender. Study characteristics, including study type, year of publication, and authorship, and the number of patients in the study were collected. Clinical data regarding presenting symptoms, location of the fibrosarcoma, use of chemotherapy and/or radiation, surgical interventions, and histopathology characteristics were gathered. Comments regarding patient outcomes were collected including survival and mortality. The collected data were then organized into graphical figures and tables.

Results

General Overview

A preliminary search yielded 632 articles after duplicates were removed, with 26 ultimately meeting inclusion criteria and were included in our study. No previous systematic reviews related to fibrosarcomas of the paranasal sinuses were identified. Articles ranged in publication date from 1952 to 2019. A total of 109 patients were derived from 19 case reports, four case series, and three retrospective cohort studies. All included studies are listed in Table 1.

**Table 1:** Characterization and study type of published fibrosarcoma literature

Title	Author	Year	Study type	Level of evidence	No. of patients
A case of fibrosarcoma of the ethmoid	Stratton [10]	1953	Case report	IV	1
Fibrosarcoma of the ethmoid	Prasad and Kanjilal [11]	1969	Case report	IV	1
Fibrosarcoma of the nose and paranasal sinuses	Richardson and Maguda [12]	1970	Case report	IV	2
Fibrosarcoma of nose and paranasal sinuses	Agarwal et al. [13]	1980	Case series	IV	2
Fibrosarcomas of the nose and paranasal sinuses	Broniatowski and Haria [14]	1981	Case report	IV	2
Fibrosarcoma of the maxillary sinus	Oppenheimer and Friedman [15]	1988	Case report	IV	1
Fibrosarcoma of the ethmoid	Smith and Soames [16]	1989	Case report	IV	1
Fibrosarcoma of the nose and the paranasal sinuses	Olekszyk et al. [17]	1989	Case report	IV	1
Fibrosarcoma arising in the maxillary sinus: CT and MR features	O'Connell et al. [18]	1996	Case report	IV	1
Inverted papilloma-like sinonasal epithelial hyperplasia, overshadowing underlying sinonasal fibrosarcoma: a diagnostic pitfall	Maly et al. [19]	2006	Case report	IV	1
Fibrosarcoma of the maxillary sinus	Mansouri et al. [20]	2006	Case report	IV	1
Sinonasal fibrosarcoma: a case report	Plaza et al. [21]	2006	Case report	IV	1
Unusual synchronous presentation of maxillary sinus fibrosarcoma and gemistocytic astrocytoma with a complication called leukocytoclastic vasculitis: a case report	Cadir et al. [22]	2009	Case report	IV	1
A rare sinonasal neoplasm: fibrosarcoma	Bercin et al. [23]	2011	Case report	IV	1
Infantile fibrosarcoma of the maxillary sinus: significant response	Palacios and Lam [24]	2012	Case report	IV	1
Infantile fibrosarcoma of ethmoid sinus, misdiagnosed as an adenoid in a 5-year-old child	Geramizadeh et al. [25]	2015	Case report	IV	1
Destructive fibrosarcoma of the maxillary sinus	Ekinci et al. [1]	2018	Case report	IV	1
Recurrent fibrosarcoma of maxillary sinus involving the cranial base: a rare case	Jin et al. [6]	2018	Case report	IV	1
Fibrosarcoma of the ethmoid sinus: a rare entity	Zouhair et al. [26]	2019	Case report	IV	1
Chart review					
Fibrosarcoma of the paranasal air sinuses	Cronin [27]	1973	Case series	IV	3
Unusual malignant tumours of the maxillary sinuses	Wolfowitx and Schmaman [28]	1975	Case series	IV	1
Fibrosarcoma of the nose and paranasal sinuses	Rockley and Liu [29]	1986	Case series	IV	9
Sarcomas of nasal cavity and paranasal sinuses: chondrosarcoma, osteosarcoma and fibrosarcoma	Koka et al. [30]	1994	Retrospective cohort	IIB	14
Malignant tumors of the sinonasal tract in the pediatric population	Yi et al. [31]	2012	Retrospective cohort	IIB	2
Sinonasal fibrosarcoma: analysis of the Surveillance, Epidemiology, and End Results database	Patel et al. [3]	2015	Retrospective cohort	IIB	51
Fibrosarcoma arising in the paranasal sinus: a clinicopathological and radiological analysis	Zeng et al. [5]	2018	Case series	IV	7

Clinical Characteristics Analysis

**Figure 2:**
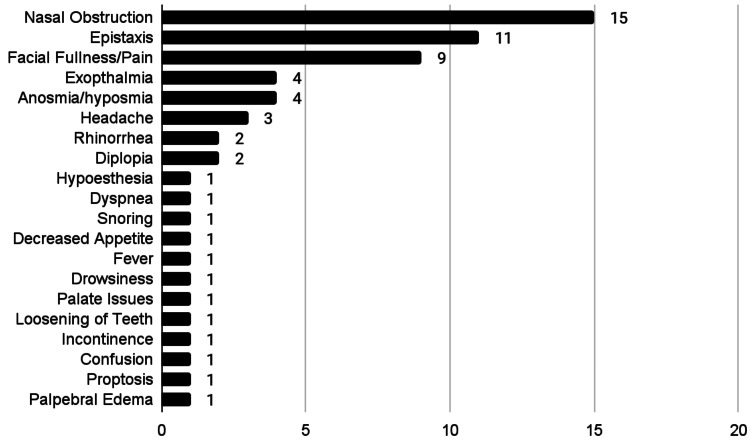
Presenting symptoms

**Figure 3:**
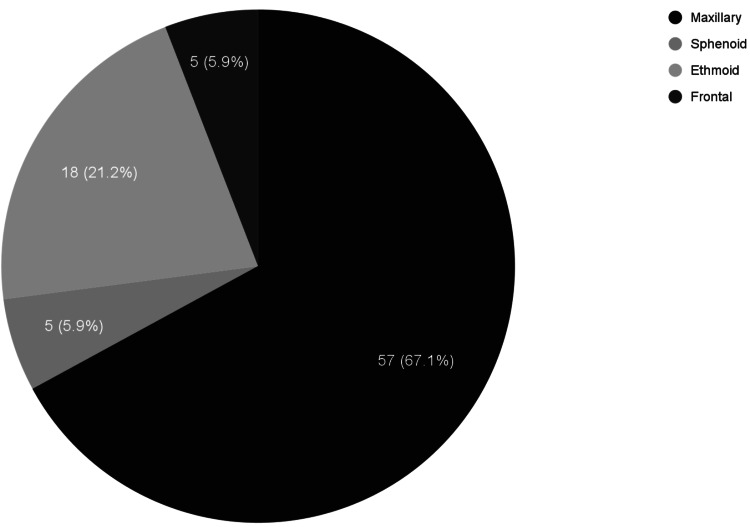
Sinuses involved

The described data for clinical characteristics can be found in Table 2. In total, 26 studies were used for the analysis of clinical characteristics. Out of 26 studies, nasal obstruction (n = 15) was noted to be the most common presenting symptom, followed by epistaxis (n = 11), facial fullness/pain (n = 9), exophthalmia (n = 4), anosmia (n = 4), headache (n = 2), rhinorrhea (n = 2), diplopia (n = 1), hypoesthesia (n = 1), dyspnea (n = 1), snoring (n = 1), decreased appetite (n = 1), fever (n = 1), drowsiness (n = 1), palatal discomfort (n = 1), loosening of teeth (n = 1), incontinence (n = 1), confusion (n = 1), proptosis (n = 1), and palpebral edema (n = 1). This information is also graphically depicted in Figure 2. Out of 26 studies, the maxillary sinus was the most common location for fibrosarcoma (n = 57), followed by the ethmoid sinus (n = 18), frontal sinus (n = 5), and sphenoid sinus (n = 5). Information regarding location is also represented in Figure 3. The provided histological information is also noted in Table 2.

**Table 2:** Overview of age, gender, symptoms, laterality, location of sinus, and histological remarks SMA: smooth muscle actin.

Author	Year	Age	Gender	Symptoms	Laterality	Sinus	Histopathology remarks
Zouhair et al. [26]	2019	13	Male	Exophthalmia	N/A	Ethmoid	Not described
Cadir et al. [22]	2009	48	Male	Facial fullness/pain	Left	Maxillary	Vimentin positive and SMA negative
Ekinci et al. [1]	2017	55	Male	Epistaxis and nasal obstruction	Right	Maxillary	Atypical fusiform cells, SMA positive, CD99, CD34, desmin, S100 myogenin, and myosin negative
Geramizadeh et al. [25]	2015	5	Male	Epistaxis, hypoesthesia, dyspnea, snoring, and decreased appetite	N/A	Ethmoid	Spindle-shaped cells, vimentin positive, and desmin negative
Jin et al. [6]	2018	45	Male	Facial fullness/pain	Right	Maxillary	Fusiform spindle cells, strong positivity for CD31 and vimentin
Maly et al. [19]	2006	52	Female	Facial fullness/pain, epistaxis, and nasal obstruction	Right	Maxillary	Spindle cells noted, positive for vimentin. Mildly positive for MIB-1
Mansouri et al. [20]	2006	16	Male	Facial fullness/pain, epistaxis, nasal obstruction, left-sided anosmia, intermittent paranasal sinus drainage, and intact left facial nerve	Left	Maxillary	Elongated spindle cells arranged in bundles
O'Connell et al. [18]	1996	36	Female	Facial fullness/pain, epistaxis, nasal obstruction, and exophthalmia	Right	Ethmoid and maxillary	Not described
Palacios and Lam [24]	2012	2.8	Female	Not described due to the patient's age	Right	Maxillary	Inter-weaving bundles of spindle-shaped cells were noted. Positive for vimentin
Plaza et al. [21]	2006	58	Male	Epistaxis, nasal obstruction, exophthalmia, rhinorrhea, hyposmia, and frequent sinus cephalalgias. A physical exam revealed palpebral hematoma, left proptosis, and orbital cellulitis	Not described	Ethmoid	Composed of elongated spindle-shaped cells arranged in a herringbone pattern
Stratton [10]	1953	56	Male	Facial fullness, nasal obstruction, drowsiness, pyrexia, bilateral pain sinusitis, headaches, and occasional incontinence	Right	Ethmoid with metastasis to the antrum, ethmoidal sphenoidal sinus, and the floor of the frontal sinus	The tissue was found to be spindle cell-like
Smith and Soames [16]	1989	24	Male	Epistaxis and anosmia	Left	Ethmoid	Cellular spindle cell tumor with varying amounts of the intercellular collagenous stroma
Agarwal et al. [13]	1980	42, 45	Female, male	Epistaxis and nasal obstruction	Left	Maxillary	Malignant cells running in various planes were described, in a crisscross pattern. Nuclei were elongated
Broniatowski and Haria [14]	1981	47, 68	Male, male	Epistaxis and nasal obstruction	Left	Maxillary	Composed of irregularly arranged, moderately pleomorphic oval cells with an interlacing pattern
Olekszyk et al. [17]	1989	73	Female	Nasal obstruction	Right	Ethmoid, maxillary, and sphenoid	Spindled neoplastic cells with an island of bone and respiratory tract epithelium with an underlying spindled neoplasm
Oppenheimer and Friedman [15]	1988	29	Male	Facial fullness/pain, sinusitis, and tenderness over the right maxilla	Right	Maxillary and sphenoid	Not described
Prasad and Kanjilal [11]	1969	8	Male	Nasal obstruction	Left	Ethmoid	The tumor consists of interlacing sheets of spindle-shaped cells with pleomorphic large irregular hyperchromatic nuclei
Zeng et al. [5]	2018	22, 41, 48, 25, 50, 43, 73	Female, female, female, female, female, male, male	Facial fullness/pain, epistaxis, and nasal obstruction	Right maxillary sinus (x4), left maxillary sinus(x2), ethmoid sinus (x1)	Maxillary (x6), ethmoid (x1)	5 well-defined and 2 ill-defined tumors. low-grade (n = 3), intermediate grade (n = 1), high grade (n = 3)
Yi et al. [31]	2012	1 9	N/A	Facial fullness/pain and nasal obstruction	Not described	Maxillary sinus (x2), ethmoid (x1)	Not described
Patel et al. [3]	2015	N/A	N/A	Not described	Not described	Maxillary (x28), ethmoid (x6), frontal (x2), sphenoid (x2)	6 well differentiated, 22 moderately differentiated, 4 poorly differentiated, 5 undifferentiated, anaplastic, 14 unknown
Wolfowitx and Schmaman [28]	1975	23	Female	Facial fullness/pain, nasal obstruction, exophthalmia, and confusion	Left	Ethmoid and maxillary	Not described
Cronin [27]	1973	2, 33, 55	Female, female, male	Supra-orbital pain with swelling over the left eye	Left	Frontal	Long spindle-shaped cells loosely arranged in a non-staining matrix
Rockley and Liu [29]	1986	N/A	N/A	Nasal obstruction and blood-stained nasal discharge; other complaints included swelling and ulceration of the palate, loosening of teeth, and swelling of the cheek of diplopia	Not provided	Fronto-ethmoid (x1), maxillary (x8)	Not described
Bercin et al. [23]	2011	47	Female	Epistaxis, nasal obstruction, diplopia, proptosis, hyposmia, headaches, and palpebral edema	Bilateral	Ethmoid, frontal	Weakly positive for CD34 and SMA

Type of Treatment Analysis

The aggregate data for treatment and outcome of patients with fibrosarcoma can be found in Table 3. There were 109 patient cases that were analyzed in regards to the type of treatment they received. In total, 46% (n = 49) of patients underwent only surgical management. In total, 39% (n = 41) of patients received radiation as a form of treatment, 10% (n = 11) of patients underwent chemotherapy and no radiation, and 5% (n = 5) of patients received both chemotherapy and radiation. Out of 52 patient cases that reported mortality information within one year of treatment, 35% (n = 18) of cases reported patient death within one year. The individual surgical approaches are described in Table 3.

**Table 3:** Treatment methods and outcomes

Author	Year	Chemotherapy or radiation	Surgical remarks	Patient outcome remarks	Reported mortality and survival
Stratton [10]	1953	The patient received chemotherapy and radiation	Surgical summary: malignant structures were removed from the antrum, ethmoids, sphenoid, and the frontal sinus as tumors were also found there	The patient tolerated the surgery well, with occasional complaints of headaches	Still alive at 6-year follow-up
Prasad and Kanjilal [11]	1969	The patient received radiation	Surgical summary: malignant structures were removed through the mouth and nose under general anesthesia	The patient tolerated the surgery well and did not complain of any symptoms. Radiological images showed normal air shadows, indicating nasal and sinus cavities were clear of obvious malignancy	N/A
Richardson and Maguda [12]	1970	1 patient case received radiation and 1 patient case did not receive chemotherapy or radiation	Surgical summary: Case 1 - ethmoidectomy was performed. Case 2 - the patient was advised to have a radical resection of the left antrum, ethmoids, and orbital exenteration. However, the patient refused orbital surgery, so only a partial left maxillectomy was done	Case 1 - the patient died 4 years after the initial diagnosis due to hemorrhage from fibrosarcoma, with local extension into the paranasal sinuses. Case 2 - tolerated surgery well without evidence of local recurrence or metastasis	Case 1 - died 4 years after initial presentation. Case 2 - was still alive at 10 months follow-up
Wolfowitx and Schmaman [28]	1975	High-grade tumor, the patient received palliative radiotherapy	No surgical intervention was done	There was no improvement after palliative radiotherapy and the patient died 3 weeks after admission	Died 3 weeks after admission
Agarwal et al. [13]	1980	The patient received radiotherapy	Surgical summary: using a Caldwell-Luc approach, resection of the tumor was done	The patient tolerated the surgery well and was noted of doing well without evidence of recurrence	Case 1 - the patient is still alive 5 years after the initial visit. Case 2 - the patient is still alive 4 years after the initial visit
Broniatowski and Haria [14]	1981	1 patient case received chemotherapy and radiation and 1 patient case did not receive chemotherapy or radiation	Surgical summary: left radical maxillectomy with ethmoidectomy, sphenoidotomy, and exenteration of the orbit was performed	Case 1 died six months after surgery from disseminated disease. At the time of his death, there had been no recurrence of the maxillary tumor. Case 2 developed local palatal recurrence, which was treated by wide excision. The patient died two weeks later from widespread disease	Case 1 -died 6 months after the initial visit. Case 2 - died 3 months after the initial visit
Rockley and Liu [29]	1986	1 patient case received chemotherapy and radiation after surgery; 2 patient cases received radical radiation after surgery	Surgical summary: 7 patient cases received surgical excision as primary treatment	1 patient who received surgical excision only survived; 1 patient who received surgical excision with chemoradiation survived; 8 patients died (4 months-14 years, range of time before death)	Case 1 - died after 1.6 years. Case 2 - died after 6 years. Case 3 - died after ¾ years. Case 4 - died after 2 years (L). Case 5 - died 1.7 years later. Case 6 - alive after 14 years. Case 7 - died after 11 months. Case 8 - died after 4 months. Case 9 - died after 4 years. Case 10 - alive after 9 years
Oppenheimer and Friedman [15]	1988	The patient received radiation	Surgical summary: radical maxillectomy and orbital exenteration	The patient tolerated the surgery well; however, died 9 months after the surgery	Died 9 months after the initial visit
Smith and Soames [16]	1989	The patient did not receive chemotherapy or radiation	Surgical summary: the eye was removed en bloc with the left ethmoidectomy, with partial maxillectomy and removal of the cribriform plate and surrounding bone	The patient tolerated the surgery well; however, had intracranial edema and later had chronic osteomyelitis of the cranial bone flap. The patient made a full recovery five years later, there was no evidence of recurrence	Still alive at 5-year follow-up
Olekszyk et al. [17]	1989	The patient received radiation	Surgical summary: the patient received maxillectomy and ethmoidectomy	The patient tolerated the surgery well; however, patient mortality status was not reported	N/A
Koka et al. [30]	1994	7 patients received chemotherapy and 11 patients received radiation	Not described	Survival at 5 years was 21% of patients. Fibrosarcoma was 78% at 1 year, 2 years was 42%, 28% at 3, and 21% at 5 years. Female patients had a slightly better survival rate	78% alive after 1 year, 42% alive after 2 years, 28% alive after 3 years, 21% alive after 5 years
O'Connell et al. [18]	1996	The patient received both chemotherapy and radiation	Not described	Six weeks post-chemoradiation therapy, CT showed continued growth of the lesion, extending to the intracranial region	N/A
Maly et al. [19]	2006	The patient did not receive chemotherapy or radiation	Surgical summary: medial maxillectomy with excision of the tumor	Patient outcome not provided	N/A
Mansouri et al. [20]	2006	The patient received chemotherapy and radiation	Surgical summary: left medial maxillectomy was done	The patient tolerated the surgery well. However, 2 years after completion of radiation therapy, the patient died with evidence of metastasis	Died 2 years after the initial visit
Plaza et al. [21]	2006	The patient did not receive chemotherapy or radiation	Surgical summary: complete removal of the neoplasia was achieved via an endoscopic approach	The patient tolerated the procedure well. However, the patient died from pancreatic cancer, without evidence of metastasis on pathology reports	Died 2 years after the initial visit due to another primary cancer
Cadir et al. [22]	2009	The patient received radiation	The patient had a partial maxillectomy with orbital reconstruction	The patient tolerated the procedure well; however, was later found to have inflammatory granulation and leukocytoclastic vasculitis around the skin graft	Died 2.5 years after the initial visit
Yi et al. [31]	2012	Both patients received chemotherapy	Surgical summary: Case 1 had a wide excision of the tumor. Case 2 had excision of the tumor via open rhinoplasty approach	Patients tolerated the procedure well. Both patients had no evidence of disease after surgery and chemotherapy	Case 1 - alive at 10.4-year follow-up. Case 2 -Alive at 5.7-year follow-up
Palacios and Lam [24]	2012	The patient received chemotherapy	No surgical intervention was done	Biopsy showed eradication of tumor cells. There was no evidence of tumor recurrence at the 3-year follow-up	Alive at 3-year follow-up
Geramizadeh et al. [25]	2015	The patient did not receive chemotherapy or radiation	Surgical summary: anterior ethmoidectomy was performed with drainage of purulent material	The patient tolerated the procedure well. There were no residual tumor cells according to pathology and no adjuvant therapy was required. The patient was noted of doing well in the 6-month follow-up	Alive at 6-month follow-up
Patel et al. [3]	2015	Out of 51 patients, 30 patient cases received only surgery, 3 patient cases only received radiotherapy, and 16 patient cases received surgery and radiotherapy	Not described	Individual cases are not described. Patients treated with surgery alone (in The Surveillance, Epidemiology, and End Results database) have the greatest disease-specific survival potentially due to lower-stage disease	57.7% overall survival of maxillary sinus cases
Ekinci et al. [1]	2017	The patient received radiation	Surgical summary: total excision with the Denker approach was completed. Inferior and medial conchas were excised and medial maxillectomy was performed	The patient tolerated the surgery well. No postoperative complications were reported and the patient was discharged 3 days after surgery	N/A
Zeng et al. [5]	2018	2 patient cases received radiation	Surgical summary: radical surgical resection was done in all seven patients, including total maxillectomy (n = 6) and lateral rhinotomy (n = 1)	2 patient cases experienced local recurrence. 1 patient case died of the uncontrolled recurrent lesion and systemic failure during a 54-month follow-up period, and 2 patient cases remained stable. 1 patient case experienced tumor recurrence and tumor metastasis. 1 patient case was not reported due to loss of follow-up	Case 1 - 5.5 years later alive but with progressive disease. Case 2 - 3.25 years later dead. Case 3 - 5.4 years later alive. Case 4 - 3.1 years later alive. Case 5 - 4.8 years later alive. Case 6 - 1.2 years later death
Jin et al. [6]	2018	The patient received chemotherapy	Surgical summary: total right maxillectomy was performed. The tumor was excised along with the infiltrated right masseter, as well as the medial and lateral pterygoids	Patient experienced recurrence of tumor after 5 months	N/A
Zouhair et al. [26]	2019	The patient did not receive chemotherapy or radiation	Surgical summary: total excision of the tumor was performed endoscopically	The patient tolerated the procedure well. No residual tumor cells were found after one year of follow-up	N/A
Bercin et al. [23]	2011	The patient did not receive chemotherapy or radiation	Surgical summary: Lynch incision was performed to remove the tumor because the frontal sinus was already eroded	The patient tolerated the procedure well. There was no sign of recurrence during 2-year follow-up	Still alive at 2-year follow-up

Discussion

To the best of our knowledge, no comprehensive review of PNFS exists. Consequently, a consensus on PNFS demographics, presentation, diagnosis, treatment, and prognosis has not been found. This is significant because only a few cases of PNFS have been reported since the 1950s and an early diagnosis is associated with a more favorable outcome. We include our recommendations below.

Demographics and Symptoms

Cancer of the paranasal sinuses is a rare condition alone, with one case occurring in every 100,000 people. Studies have shown that paranasal sinus tumors tend to occur at an average age between 50 and 60 years [32]. Paranasal sinus cancers from 1999 to 2007 were seen to occur twice as high in males than females [33], while we saw an equal representation of female and male presentations for fibrosarcomas of the paranasal sinuses. Our review found that the most common site for fibrosarcoma of the sinuses was the maxillary sinus, which could be due to the fact that the maxillary sinus is the largest paranasal sinus.

In our review, the most common presenting symptom was nasal obstruction followed by epistaxis and facial fullness/pain. These symptoms are consistent with other cancers of the paranasal sinus. These presenting symptoms are common with many other conditions and can oftentimes be overlooked. The presence of unilateral symptoms that do not improve with treatment should raise the suspicion of a possible mass such as a fibrosarcoma and should warrant further workup [33].

Diagnostic Methods

In routine clinical practice, paranasal fibrosarcoma is commonly misdiagnosed as other neoplasms due to its rarity and non-specific symptomatology. Therefore, it is critical to be familiar with the imaging features that differentiate paranasal fibrosarcoma from other malignancies [5]. Additionally, the rarity of the disease and the relatively few studies within the literature examining the imaging characteristics of PNFS continue to make the preoperative diagnosis of PNFS a challenge [5]. It is also critical that physicians be aware of the advantages and disadvantages of the different imaging modalities. The most common diagnostic method to evaluate a mass of the paranasal sinus area is nasal endoscopy [31]. Histology can additionally aid in further diagnosis. Histological characteristics for fibrosarcoma tend to be consistent with spindle cells that are often arranged in a herringbone pattern with staining for CD34 and vimentin [34], which was seen across many of our cases as seen in Table 2.

When suspecting PNFS, there are particular findings found on certain modalities that may rule in or out other diagnoses on the differential [35]. One study identified the common CT and MRI features seen in patients with confirmed PNFS [5]. This particular study confirmed that PNFS commonly presents as a solitary lobulated or irregular heterogeneous mass, with either well- or ill-defined margins. Furthermore, there should be increased suspicion of PNFS when the well- or ill-defined paranasal neoplasm appears mildly hypointense on T2-weighted MRI that also shows bone destruction and a heterogeneous delayed contrast enhancement pattern [5]. These common features of PNFS emphasize the importance of radiographic findings to arrive at the diagnosis of this already rare and complex disease process.

Treatment and Mortality

An accepted mainstay treatment of fibrosarcoma of the sinuses currently does not exist. It is important to recognize that local recurrence is common for PNFS, and distant metastasis rarely occurs as well. According to analyzed studies, the most commonly used type of treatment was surgical management, as shown in Table 3. This could be due to the local destructive feature of fibrosarcomas as opposed to a metastatic nature [22]. Some studies suggest radiotherapy along with surgical management improves survival rates [22]. While other studies recommend surgery as the mainstay of treatment, with radiotherapy for more malignant tumors, as reflected by the results of this study [23,26]. Due to the frequent local recurrent nature of fibrosarcomas, some studies recommended a wide local excision with an extensive surgical border [1,14,22]. It is also worth mentioning the use of endoscopic surgery as opposed to open surgeries. While some studies suggest the use of endoscopic surgeries due to the decreased surgical complications and length of stay, other studies disapprove of the use of endoscopic surgeries due to the extensive regional nature of the tumor.

Additional research is required to give a definitive statement on indications and contraindications in the use of endoscopic surgeries in fibrosarcoma removal. According to the analyzed studies, it is suggested that surgeons are opting to mainly treat with surgical management as opposed to a multimodal type of management. We recommend that surgeons approach fibrosarcomas with local excision and large surgical border, followed by chemotherapy and radiotherapy, especially if the tumor cannot be fully removed or if surgical border involvement on pathology report is revealed. In addition, for tumors that are unresectable, it is suggested that preoperative chemotherapy should be used to decrease tumor size followed by resection.

Our results suggest that PNFS is associated with a high rate of mortality, with death occurring within one year of treatment in 35% (n = 18) of cases. However, mortality information was only available for 47% (n = 52) of patient cases. Studies that include long-term patient outcomes are needed to better assess PNFS prognosis. Published data have suggested that prognosis is associated with the degree of histological differentiation [18]. Similarly, additional studies on the rate of PNFS reoccurrence are needed. Past reviews have suggested a high rate of PNFS reoccurrence, but the studies in this review did not include sufficient prognostic data [3]. Once these data are included, conclusions on which treatments are associated with favorable outcomes can be made.

Limitations

There are several notable limitations worth mentioning. Since there were no prospective studies found, all data are based on retrospective research. Additionally, the majority of studies were case studies and series, and there were no randomized controlled trials comparing various treatment approaches, making it difficult to compare the efficacies of treatment approaches. The use of solely one primary treatment was not explicitly in the articles, which may skew the results. The mortality data were gathered from sources that mentioned any mortality information within the article. Since timeframes varied greatly for reporting the death and postoperative prognosis of patients, the mortality results may be skewed. Furthermore, the reporting of larger studies oftentimes grouped varying tumors as well as locations other than the paranasal sinuses in analysis, making some data difficult to extract. Across studies, there was not a uniform way in which data were presented, causing some information to be unavailable. To address these limitations, it would be important for larger and more standardized studies to further support our findings. Furthermore, more consistent reporting of patient progress following treatment will help with the assessment of optimal treatment options.

## Conclusions

Fibrosarcoma of the paranasal sinuses is a rare but dangerous disease. By conducting this study, we aim to provide physicians with a comprehensive review to assist in the management of PNFS. Physicians should maintain a high index of suspicion when presented with a patient with non-specific symptoms, such as nasal obstruction and facial fullness and pain, which are unresolved. Nasal endoscopy can be utilized to visualize the mass, and the use of CT and MRI can aid in further diagnosis. Ultimately, histology can confirm the final diagnosis. The mainstay of treatment is surgical excision with the use of radiation or chemotherapy depending on resectability and surgical borders. With a high rate of mortality, early identification and treatment are essential.

## Appendices

Search terms

Embase search: ('fibrosarcoma'/exp OR fibrosarcoma OR 'fibroblastic sarcoma'/exp OR 'fibroblastic sarcoma' OR (fibroblastic AND ('sarcoma'/exp OR sarcoma))) AND ('sinus'/exp OR sinus OR paranasal OR 'maxillary'/exp OR maxillary OR frontal OR ethmoid OR 'sphenoid'/exp OR sphenoid).

PubMed and Cochrane search: (fibrosarcoma OR fibroblastic sarcoma) AND (sinus OR paranasal OR maxillary OR frontal OR ethmoid OR sphenoid).
